# Sustainable Recovery of Phlorotannins from *Durvillaea incurvata*: Integrated Extraction and Purification with Advanced Characterization

**DOI:** 10.3390/antiox14030250

**Published:** 2025-02-21

**Authors:** Pamela Raquel Rivera-Tovar, Gabriela Contreras-Contreras, Paulina Isabel Rivas-Reyes, Jara Pérez-Jiménez, Maximiliano Martínez-Cifuentes, José Ricardo Pérez-Correa, María Salomé Mariotti-Celis

**Affiliations:** 1 Nutrition and Dietetics School, Faculty of Medicine, Universidad Finis Terrae, Pedro de Valdivia 1509, Providencia, Santiago 7501015, Chile; privera@uft.cl (P.R.R.-T.); gabriela.contreras@ug.uchile.cl (G.C.-C.); 2 Food Technology Section, Department of Analytical Chemistry, Nutrition and Food Science, School of Veterinary Sciences, University of Santiago de Compostela, 27002 Lugo, Spain; privasr@uft.edu; 3 Department of Metabolism and Nutrition, Institute of Food Science, Technology and Nutrition (ICTAN-CSIC), 28040 Madrid, Spain; jara.perez@ictan.csic.es; 4 CIBER de Diabetes y Enfermedades Metabólicas Asociadas (CIBERDEM), Instituto de Salud Carlos III (ISCIII), 28029 Madrid, Spain; 5 Departamento de Química Orgánica, Facultad de Ciencias Químicas, Universidad de Concepción, Edmundo Larenas 129, Concepción 4070371, Chile; maxmartinez@udec.cl; 6 Chemical and Bioprocess Engineering Department, School of Engineering, Pontificia Universidad Católica de Chile, Vicuña Mackenna 4860, Macul, Santiago 7820436, Chile; jperezc@uc.cl

**Keywords:** brown seaweed, integrated extraction–purification processes, spectrophotometric, UHPLC-QToF MS/MS, mass balance

## Abstract

The rising demand for bioactive compounds from marine resources highlights the need for sustainable separation technologies. This study introduces an integrated process combining ultrasound-assisted extraction (USAE) and resin purification (RP) to isolate phlorotannins from *Durvillaea incurvata*, a brown seaweed with significant biomedical potential. Using a 32.5% ethanol–water solvent system for USAE followed by RP on Diaion HP-20 resin, phlorotannins were enriched 2.4-fold, with simultaneous removal of interfering compounds such as mannitol (~100%), which was demonstrated by FTIR and HPLC-IR analysis. Advanced characterization using UHPLC-QToF-MS/MS identified five novel phlorotannins with polymerization degrees of 3 to 8 phloroglucinol units in both USAE extracts and post-RP. Mass balance based on spectrophotometric measurements indicated a purification factor of ~2, confirming process effectiveness. RP streams showed distinct phlorotannin profiles, with one phlorotannin exceeding 70% relative abundance. However, MS/MS results showed significantly lower recoveries than spectrophotometric data, revealing a novel insight into RP purification. These findings highlight the critical role of comprehensive chemical characterization in optimizing sustainable phlorotannin extraction from seaweed. They propose a framework for scalable, eco-efficient technologies for achieving high-purity phlorotannin recovery. This approach facilitates the development of phlorotannin-based applications in the nutraceutical and pharmaceutical industries.

## 1. Introduction

Researchers have described various forms of nutritional supplementation to prevent and control oxidative stress-induced pathologies [1]. Among various bioactive compounds, attention has also focused on polyphenols, a wide range of plant-based metabolites with multi-target biological functionality [2,3,4,5,6]. Phenolic compounds are not limited to the plant kingdom; they are also present in seaweeds—particularly brown seaweed, which contains phlorotannins [7].

Phlorotannins are phenolic compounds constituted by phloroglucinol (1,3,5-trihydroxybenzene) chains and net-like structures of diverse molecular weights. They are classified into six groups, according to the type of linkages between phloroglucinol units and their content of hydroxyl groups: (i) fucols, with aryl–aryl linkages; (ii) phlorethols, with aryl–ether linkages; (iii) fucophlorethols, with aryl–aryl and aryl–ether units; (iv) fuhalols, with aryl–ether linkages and additional OH groups in every third ring; (v) carmalols and (vi) eckols, both with dibenzodioxin linkages [8]. Eckols differ from carmalols by their low molecular weight and by the presence of a phenoxyl substitution at the C4 position, while in carmalols, the phenoxyl substitution occurs at the C3 position [8]. These highly reactive metabolites have been pointed out, in in vitro and preclinical studies, as potent modulators of various biochemical processes linked to the prevention and treatment of major chronic diseases such as cancer [9], diabetes type II [10], neurodegenerative disorders [11], among others. These conditions are frequently linked to increased oxidative stress and inflammatory responses [12,13]. The anti-inflammatory effects of phlorotannins have been reported in extracts rich in carmalols, fucols, and eckols, demonstrating efficacy in both cell-based and animal models [12,14,15]. And, notably, in a clinical trial involving healthy adults supplemented with capsules containing ethanolic extracts from brown seaweed, it was found that high-molecular-weight phlorotannins were metabolized and absorbed primarily in the large intestines, leading to a reduction in inflammation biomarkers [12].

Indeed, the magnitude of the activity of the brown seaweed extracts/fractions is significantly different from that of the individual phlorotannins contained therein, which makes fully characterized high-purity extracts novel candidates for developing natural therapeutic alternatives with potential medical applications [16]. Therefore, selective separation techniques are research areas of interest [10,17]. The sustainable extraction of phlorotannins from *Durvillaea incurvata*, a brown seaweed highly available on the Chilean coast, is an attractive alternative to scale up the production of phlorotannin-rich extracts [10,16,18]. The frond of this seaweed has shown a significantly higher total polyphenol content compared to those of other brown seaweed species, such as *Lessonia spicata* [10], making it a valuable source of bioactive compounds. Additionally, *Durvillaea incurvata* is highly abundant on the Chilean coastline (National Landing 2022: 11,179 tons, Sernapesca), where its excessive accumulation, exacerbated by climate change and oceanographic changes such as warmer temperatures and altered currents, has increasingly impacted coastal ecosystems. This accumulation negatively affects tourism and economic activities by altering beach aesthetics and emitting unpleasant odors as the biomass decomposes [19,20,21]. Sustainable valorization strategies, such as the extraction of phlorotannins, offer an opportunity to mitigate these negative effects while supporting the development of high-value products from this abundant marine resource.

Hot Pressurized Liquid Extraction (HPLE) has been one of the most employed environmentally friendly techniques for obtaining phlorotannin-rich extracts efficiently [10,18], although its scaling to commercial production is still challenging [22]. In turn, Ultrasound-Assisted Extraction (USAE) is a scalable technology that effectively recovers these bioactive compounds but, like HPLE, lacks selectivity [17,23]. Recent studies have shown that *Durvillaea incurvata* phlorotannin extracts obtained through HPLE are rich in mannitol. This sugar alcohol has been shown to preserve the antioxidant properties of various terrestrial plant extracts subjected to spray drying due to its thermoprotective effect [24,25]. However, this effect has been questioned because mannitol can also scavenge free radicals, leading to overestimating antioxidant capacity and total polyphenol measurements after spray drying [26].

Integrating selective purification methods following the extraction process appears to be an effective approach for obtaining phlorotannin-rich extracts with fewer interferents, thereby enhancing their bioactive potency [27]. Additionally, the analysis of purified extracts is simpler, leading to a deeper understanding of their chemical composition, which in turn enables more effective exploitation of their therapeutic potential.

Colorimetric techniques allow the determination of seaweed extracts’ total phenolic compounds and antioxidant capacities. However, the complex polymeric structure and similar polarity of phlorotannins make them difficult to separate and quantify. In this sense, the continuous development of advanced instrumental techniques opens the possibility of unveiling the occurrence of moieties (bonds and functional groups). In this way, different mass spectrometry (MS) approaches, with or without coupling to liquid chromatography systems, have allowed the analysis of phlorotannin-rich extracts of diverse species of brown seaweed. Systems coupled with LC based on different analyzers are valid for detecting phlorotannins of low molecular weight [28]. At the same time, matrix-assisted laser desorption/ionization–time-of-flight (MALDI-ToF) can ionize and analyze larger compounds of up to 27 degrees of polymerization (DP) [28]. Also, Fourier transform infrared spectroscopy (FTIR) is a highly effective qualitative technique for identifying potential bonds and functional groups present in a sample, indicating the presence of any specific species of interest [29]. It has been suggested as a valid tool for characterizing polyphenol profiles in brown seaweed extracts [30]. However, due to the chemical complexity of phlorotannins, new analytical approaches need to combine the mentioned techniques, which allow their routine characterization, for instance, in the context of nutraceutical development.

The Chilean coast is rich in seaweed, and several studies have explored their phlorotannin profiles. Ethanolic extracts obtained under atmospheric conditions from three Chilean brown seaweed (*Durvillaea antarctica*, *Lessonia spicata*, and *Macrocystis integrifolia*) and analyzed by LC-DAD-ESI-MS/MS (Liquid Chromatography–Diode Array Detector–Electrospray Ionization–Tandem Mass Spectrometry) showed differential profiles regarding the DP of these compounds, e.g., trimers to octamers in *D. Antarctica* and trimers to tetramers in *L. spicata* [31]. Recently, phlorotannins from *Durvillaea incurvata* and *Lessonia spicata* extracts obtained by HPLE-RP (Hot Pressurized Liquid Extraction–Resin Purified Process) were characterized using liquid UHPLC-Orbitrap (Ultra-High-Performance Liquid Chromatography) and UHPLC-QoF-MSn (Ultra High-Performance Liquid Chromatography–Quadrupole Time of Flight–Tandem Mass Spectrometry with Multiple Stages) techniques [27]. Fucols, phlorethols, and fucophlorethols isomers up to 4 DP were the most representative phlorotannins in the extracts of both Chilean seaweeds. Eckols, carmalols, fuhalols, phenolic acids, and flavonoids were also detected.

Interestingly, the analysis of *Durvillaea incurvata* extracts showed low abundances of high molecular weight phlorotannins for the first time (11–21 DP) [27]. The differences in the metabolite profile of evaluated Chilean brown seaweed extracts indicate that achieving a deeper molecular and structural characterization of these compounds is essential to elucidate the mechanisms behind their selective separation. Moreover, this becomes even more crucial when considering scaling up their production using sustainable technologies.

Mass balances are essential for analyzing performance and designing integrated extraction and purification processes, particularly when dealing with complex streams [32]. However, few studies have utilized mass balances to assess the efficiency of integrated processes aimed at producing purified, polyphenol-rich extracts from natural sources. Mass balance analysis is especially challenging in the case of phlorotannin separation processes, as their identification and quantification are complicated by both the structural complexity of phlorotannins and the limited availability of analytical standards.

In the specific case of resin purification (RP) by adsorption, each outlet stream exhibits a distinct polyphenolic profile [33], resulting in a unique spectrum of bioactivities for each stream. Without a comprehensive mass balance analysis, it is difficult to accurately evaluate the overall performance of the integrated extraction and purification process or assess the potential of each outlet stream for use in natural therapeutic applications.

The objectives of this study were to develop an integrated water–ethanol-based USAE-RP process for the selective separation of phlorotannins from Durvillaea incurvata, characterize the extracted compounds using spectrophotometric analysis, HPLC-IR, FTIR, and UHPLC-QToF MS/MS, and assess the efficiency and selectivity of the process through a combination of parameters (mass balance analysis, evaluating yield, recovery rates, mannitol removal efficiency, purities, and purification factors). This integrative approach highlights the potential of marine resources for bioactive compound recovery and establishes a foundation for optimizing green separation technologies in natural alternatives with potential medical applications derived from *Durvillaea incurvata*.

## 2. Materials and Methods

### 2.1. Seaweed Material

Brown seaweed frond (*Durvillaea incurvata*) samples were collected from Concepción, Chile (36°49′37.2″ S 73°2′59.2″ W) in the fall of 2023. The samples were washed with water, cut, frozen, and freeze-dried. Then, they were ground and sieved (Tyler sieve n°25, <710 µm) and stored in dark polyester bags at 20 °C. The proximal composition of the raw material was determined using standard analytical procedures [34], among them moisture (10.90 ± 0.08%), ash (19.54 ± 0.03%), crude protein (0.82 ± 0.10%), crude fat (0.41 ± 0.03%), crude fiber (0.98 ± 0.25%), and carbohydrates by difference (67.36 ± 0.14%).

### 2.2. Chemicals

Analytical grade solvents (acetone, acetonitrile, and ethanol) and reagents (2,4-dimethoxybenzaldehyde [DMBA], Folin–Ciocalteu phenol reagent, HCl, H_3_PO_4_, NaOH, and the salts K_2_HPO_4_, KH_2_PO_4_, and Na_2_CO_3_) were acquired from Merck, Germany. Additional materials, including macroporous resins (Diaion HP-20), fluorescein, 2,2′-Azobis(2-amidinopropane) dihydrochloride (AAPH), and the standards Trolox, phloroglucinol, gallic acid, and mannitol were purchased from Sigma-Aldrich, Saint Louis, MI, USA.

### 2.3. Extraction Techniques

#### 2.3.1. Atmospheric Solid–Liquid Extraction (ASLE)

For ASLE, 10 g of ground seaweed frond was mixed with 100 mL of 60% *v/v* acetone–water. The extraction was performed at 20 °C with constant magnetic stirring (IKA C-MAG HS, Stauffer, Germany) in a cooling tube system for 6 h. This extract was used as a reference extract (RE) since it is assumed to contain nearly 100% of the extractable polyphenols from the natural matrix [35]. The RE was used solely to calculate the recoveries of polyphenols and phlorotannins achieved by USAE and to estimate relative masses for the USAE mass balance.

#### 2.3.2. Ultrasound-Assisted Extraction (USAE)

For USAE, 20 g of ground seaweed frond was mixed with 200 mL of 32.5% *v/v* ethanol–water. The extraction process was conducted using a 1500 W ultrasonic device (BIOBASE, Jinan, Shandong, China) operating at a constant frequency of 20 kHz, a controlled temperature of 30 °C, with a jacket cooling system, 40% power, and one cycle of 30 min. Experimental conditions were established based on preliminary tests aimed at obtaining the highest phlorotannin content in the extracts. This extract was called crude extract (CE).

Following the extractions (ASLE and USAE), RE and CE were centrifuged for 5 min at 4.500 rpm (DM0412 DLAB centrifuge, Beijing, China) and subjected to rotary evaporation (RE-100 PRO DLAB, Beijing China) until dry. The resulting concentrated extracts were reconstituted with 26 mL of water and freeze-dried (BIOBASE, Jinan, Shandong, China) for 24 h. The solid powder extracts were stored in a bag in the dark until their physicochemical characterization.

### 2.4. Resin Purification (RP) Process

For RP, a column (Büchi C-690 26/100) was packed with macroporous resin (Diaion HP-20) as adsorbent, and 80% *v/v* ethanol–water was used as an elution agent [27]. In comparison to other macroporous resins, such as SP-850, XAD-2, XAD-4, and XAD-7HP, HP-20 resin has demonstrated the highest adsorption capacity for phlorotannins [36,37]. RP consisted of four stages: (i) adsorption of extract, (ii) washing with water, (iii) elution with ethanol, and (iv) regeneration of the resin with HCl and NaOH [38]. The CE and the solvents (water, ethanol, HCl, and NaOH) were pumped through the RP system at 3 mL/min using a peristaltic pump (PP-X575). The process followed this sequence: 20 mL of CE reconstituted in water (3 mg/mL), 40 mL of water, 40 mL of 80% EtOH, and finally, the regeneration consisted of passing 40 mL of water, 40 mL of HCl (2N), 40 mL NaOH (1N), and 40 mL of water [27]. Three main output streams were collected: the outlet stream during the column charging process (CS), the outlet washing stream (WS), and the elution stream (ES), which corresponds to the purified extract. These streams were subsequently freeze-dried for storage until their later analysis.

### 2.5. Chemical Analyses

#### 2.5.1. Total Polyphenol Content (TPC)

The TPC of all samples was determined spectrophotometrically using the Folin–Ciocalteu assay [28]. For analysis, samples were reconstituted (20 mg in 10 mL of water) and analyzed in duplicate following the method described in [39], with some modifications for adaptation to a 96-well microplate reader (TECAN Infinite 200 PRO MPlex, Switzerland). Aliquots of each sample, standard, or water (20 μL) were mixed with Folin–Ciocalteu reagent diluted 1:2 with water (10 μL), 150 μL of water, and 10% *w/v* aqueous solution of Na_2_CO_3_ (20 μL). The mixture was shaken and allowed to react for 1 h at room temperature (20 °C) in darkness; then, the absorbance was measured at 765 nm. Sample absorbances were interpolated against a gallic acid calibration curve (30–150 mg/L) and a phloroglucinol calibration curve (50–250 mg/L). Results are expressed as milligrams of standard equivalent (gallic acid or phloroglucinol) per gram of dried extract or RP stream.

#### 2.5.2. Total Phlorotannin Content (TPhC)

The samples were also analyzed using the DMBA method [40]. For analysis, samples were reconstituted (20 mg in 10 mL of water) and analyzed in duplicate following the method described in [40], with some modifications [41]. The reaction began with shaking for 30 min, followed by incubation at 25 °C in the dark for 60 min. Absorbances were measured at 515 nm using a 96-well microplate reader (TECAN Infinite 200 PRO MPlex, Männedorf, Switzerland). Sample absorbances were interpolated against a phloroglucinol calibration curve (1–40 mg/L). Results are expressed as milligrams of phloroglucinol equivalent (PhE) per gram of dried extract or RP stream.

#### 2.5.3. Antioxidant Capacity (AC)/Oxygen Radical Absorbance Capacity Assay (ORAC)

The ORAC assay was performed as described in [42], with some modifications [10]. In a 96-well microplate, 250 µL of 55 nM fluorescein, diluted in phosphate buffer saline (PBS) at pH 7.4 (75 mM, prepared with K_2_HPO_4_ solution and KH_2_PO_4_ solution in nanopure water), was added to 25 µL of extract, Trolox standard, or PBS (blank). The microplate was incubated for 30 min at 37 °C, and the reaction was initiated by adding 25 µL of freshly prepared 153 mM AAPH solution in PBS using an automatic injector. Fluorescence was recorded every minute for 1 h at 37 °C using a microplate reader (TECAN Infinite 200 PRO MPlex, Männedorf, Switzerland). The reference calibration curve was constructed with Trolox solutions ranging from 2 to 8 mg/L; analyses were performed in duplicate. Results are reported as micromoles of Trolox equivalents (TEs) per gram of dried extract or RP stream.

#### 2.5.4. Antioxidant Capacity (AC)/DPPH Assay

The DPPH method was performed as described by other authors [43], with modifications [10]. For analysis, samples were reconstituted (20 mg in 5 mL of water), diluted 1:6, and analyzed in duplicate. Absorbances were measured at 520 nm (Genesys 150 UV-Vis, Thermo Fisher Scientific, Waltham, MA, USA) and interpolated against a Trolox calibration curve (2–8 mg/L). Results are expressed as micromoles of Trolox equivalents (TEs) per gram of dried extract or RP stream.

#### 2.5.5. Fourier Transform Infrared Spectroscopy Analysis of USAE and RP Streams

FTIR was performed in a Spectrum Two IR spectrophotometer (Perkin Elmer, Shelton, CT, USA) with attenuated total reflection (ATR) (Pike Instruments, Madison, WI, USA) in the range of 400–4000 cm^−1^ [44]. This analysis identified functional groups in the CE and the RP output streams (CS, WS, and ES). Furthermore, the FTIR spectra of phloroglucinol and mannitol were studied to determine the selectivity of the USAE-RP process.

#### 2.5.6. Mannitol Content Determination

Mannitol was quantified in all samples using an HPLC-IR system [10]. Chromatographic analysis was performed on the Thermo Scientific Vanquish Flex HPLC system (Thermo Fisher Scientific, Waltham, MA, USA) coupled with a refractive index detector (RefractoMa 520 ERC, Thermo Fisher Scientific, Waltham, MA, USA). The autosampler was maintained at room temperature. The column used was an APS-2 Hypersil™ (150 mm × 4.6 mm, 5 µm) from Thermo Fisher Scientific (Waltham, MA, USA). The column temperature was set at 50 °C. The mobile phase consisted of 250 mM H_3_PO_4_ prepared in 80% acetonitrile. A sample volume of 20 µL was injected, and the flow rate was set at 0.3 mL/min. The total analysis time was 18 min. Mannitol quantification in the samples was carried out using a calibration curve (100 to 1000 mg/L). Analyses were performed in duplicate. Results are expressed as milligrams of mannitol (Ma) per gram of dried extract or RP stream.

#### 2.5.7. Tentative Identification of Phlorotannins Using a UHPLC Coupled with a QToF Detector

A tentative identification of the phlorotannins in all USAE-RP streams was carried out using UHPLC-QToF. Mass spectrometry analysis was conducted using a previously described methodology, with slight modifications [27].

##### Sample Injection and Chromatograph and Detector Conditions

Briefly, UHPLC-MS^2^ analysis was performed on a UHPLC-QToF system (Bruker Daltonics, Billerica, MA, USA). The autosampler was set at 4 °C, and a C18 column (2.1 mm × 100 mm, 1.7 µm) (Phenomenex, Torrance, CA, USA) was used with a temperature of 40 °C. A 5 µL sample was injected in a gradient mode, with a mobile phase A consisting of 0.1% formic acid and a mobile phase B consisting of 90% acetonitrile/0.1% formic acid. The gradient was set to start with 12% B for 1 min, then 99% B at 11 min, 99% B at 13.5 min, 12% B at 14 min, and 12% B at 15 min. The mass spectrometry data were acquired in the range 50 *m*/*z* to 1300 *m*/*z* in the negative mode. The capillary voltage was 4500 V, the nebulizer pressure was 2.5 bar, and the dry gas temperature was 250 °C, with a flow rate of 8 L/min. The collision energy was between 18 eV and 50 eV.

##### Identification of Phlorotannins

Signals corresponding to phlorotannins were identified using Compass Data Analysis 4.4 software (Bruker Daltonik, Billerica, MA, USA). MS^2^ and MS^1^ spectra were analyzed in this study to identify specific fragmentation patterns. Our analysis was focused on the MS^1^ spectra, where characteristic fragmentation patterns were observed. Phlorotannins were identified based on their [M − H]^−^ ions. This approach aligns with the methodology reported by Hyo Moon et al. [45], who successfully identified several phlorotannins using MS^1^ spectra. After tentative identification, the phlorotannins were classified according to their DP. An error of up to ±10 ppm was considered, as typically considered for phenolic compounds on a UHPLC-QToF instrument [27,46].

##### Use of Areas

Once the tentative identification and classification were made, the relative areas for the different phlorotannins were determined. The distribution of phlorotannins in the CE and each of the three RP output streams was calculated. For this, the total area of phlorotannins identified in each sample was considered to represent 100 percent for each case.

### 2.6. Statistical Analysis

The ASLE, USAE, and RP processes were performed in triplicate, and chemical analyses were performed in duplicate. The experimental results obtained are expressed as means ± SD. Statistical analysis was carried out using Minitab^®^ Statistical Software v.21. Analysis of variance (ANOVA) at a 95% confidence level (*p* < 0.05) was applied to compare all the obtained responses with Tukey’s post-hoc test after verifying the normal distribution of the data and homogeneity of variances, respectively.

### 2.7. Relative Mass Balance and Selective Separation Performance Parameters

A complementary analysis was performed using the TPC, TPhC, mannitol measurements, and the relative areas from UHPLC to perform a relative mass balance in the USAE (Equation (1)) and the RP (Equation (2)).

(1) $$m_{i_{seaweed}}=m_{i_{CE}}+m_{extraction\;cake}+e$$

(2) $$m_{i_{CE}}=m_{i_{CS}}+m_{i_{WS}}+m_{i_{ES}}+e$$

where *m* is the mass (g or mg) and the subindex *i* refers to the evaluated components (extractable solids or ExS, total polyphenols, total phlorotannins, mannitol, specific phlorotannins); *e* represents the difference between the input and output relative mass. The relative masses of the seaweed or pre-USAE (*m_i_* _seaweed_) are determined based on the reference extract (RE). The masses of ExS from CE and streams were determined by freeze-drying the respective samples. Masses of total polyphenols (TPs), total phlorotannins (TPhs), and mannitol were determined based on TPC, TPhC, and mannitol content values, respectively.

Selective separation performance parameters were calculated to determine (i) the yield achieved by USAE in terms of ExS (Equation (3)), (ii) the USAE polyphenol or phlorotannin recoveries (Equation (4)), (iii) the mannitol removal efficiency (Equation (5)), (iv) the polyphenol and phlorotannin purities (Equation (6)), and (v) the purification factor in terms of TP and TPh (Equation (7)). These parameters were determined as follows:

(3) $${yield}_{ExS}\;(\%)=m_{CE}/m_{seaweed}\times 100$$

where *m_CE_* is the dry mass of the extract generated by USAE and *m_seaweed_* is the mass of dried seaweed used in USAE.

(4) $${Rec}_{j}\;\left(\%\right)=m_{j_{CE}}/m_{j_{seaweed}}\times 100$$

where *Rec_j_* is the recovery of component *j*, which can be TP or TPh, *m_TP_* is an estimation of the TP mass as phloroglucinol equivalent from the Folin–Ciocalteu assay, and *m_TPh_* is an estimation of the TPh mass as phloroglucinol equivalent from DMBA assay.

(5) $${Eff}_{Ma}\;\left(\%\right)=m_{Ma\;removed}/m_{{Ma}_{CE}}\times 100$$

where *Eff_MA_* is the efficiency of RP to remove the mannitol contained in CE, *m*_Ma removed_ is the result of the difference between *m*_Ma CE_ and *m*_Ma purified extract_.

(6) $$P_{j}\;\left(\%\right)=m_{j}/m_{ExS}\times 100$$

where *P_j_* is the purity of component *j*, which can be TP or TPh.

(7) $$F_{Purification}=P_{TP\;ó\;TPh}\;after\;RP/P_{TP\;ó\;TPh}\;before\;RP$$

where *F*_Purification_ is the purification factor, which represents the performance of the RP to increase the purity of the component of interest (TP and TPh).

During the preparation of this section, the authors used Grammarly, DeepL, and ChatGPT v.4 to enhance the readability of this text. After using these tools, the authors reviewed and edited the content as necessary; they take full responsibility for the content of the published article.

## 3. Results and Discussion

### 3.1. Spectrophotometric Characterization and Quantification: TPC, TPhC, AC_ORAC_, and AC_DPPH_

**Figure 1:**
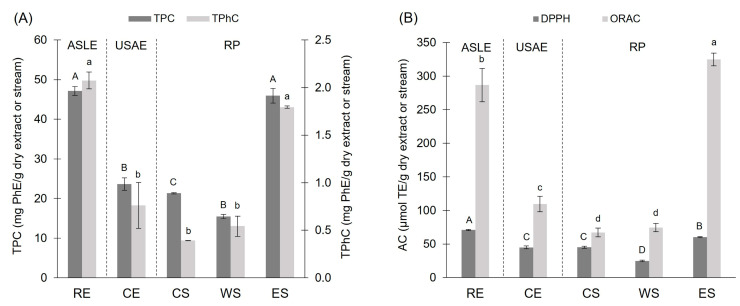
(**A**) TPC and TPhC, and (**B**) antioxidant capacity (DPPH and ORAC assays) of RE, CE, and RP streams (CS, WS, and ES) from *Durvillaea incurvata* frond. ^(A–D; a–d)^ Values that do not share a letter are significantly different.

All samples, i.e., CE and the three RP streams (CS, WS, and ES), were characterized in terms of TPC, TPhC, and AC (by ORAC and DPPH assays), as shown in Figure 1. Overall, the values determined by the Folin–Ciocalteu method were one order of magnitude higher than those obtained by the DMBA method. This discrepancy could be due to an overestimation of the Folin–Ciocalteu values caused by interference by other non-phenolic reducing compounds present in the brown seaweed (sugar alcohols, polysaccharides, pigments, and other polyphenol–protein complexes [47]) or to an underestimation of the DMBA values due to the non-detection of branched phlorotannins or those with aryl bonds or fuhalols with additional hydroxyl groups in positions 2, 4, or 6, which differ structurally from those reacting with DMBA (1,3 and 1,3,5-trihydroxybenzenes) [28]. Considering both measurement methods could offer a better approximation of seaweed polyphenol and phlorotannin quantification.

TPC of CE (23.6 ± 1.6 mg PhE/g dry extract) was slightly higher than that obtained in a previous study of *Durvillaea incurvata* frond from “Las Cruces” by HPLE (15% *v/v* glycerol; 21.1 ± 9.2 mg PhE/g dry extract). However, the absolute recovery of total polyphenols in our extract (3.6 ± 0.2 mg PhE/g dried seaweed) was approximately half the amount found in that study [10]. Although both methods (USAE-EtOH and HPLE-glycerol) produced extracts with similar polyphenol concentrations, HPLE-glycerol was more effective for total polyphenol recovery. USAE-EtOH’s lower performance may be related to the degradation of certain phlorotannins and the generation of highly reactive hydroxyl radicals in the air bubbles resulting from the acoustic cavitation of USAE [47]. Factors related to the raw material, such as harvest date, location, and pre-storage treatments, may also affect these values [48]. Nevertheless, in this case, this discrepancy in polyphenol recovery is mainly due to the extraction methods. In the previous study, the same seaweed species and anatomical part were used, and both reference extractions with ASLE achieved similar absolute yields (8.2 ± 0.2 PhE/g dry seaweed in the current study and 11.7 mg PhE/g dry seaweed in the previous study).

It is worth noting that the TPC of our CE sample expressed in GAE (12.7 ± 0.9 mg GAE/g dry extract) was practically the same as that of the optimal extract (12.8 ± 2.3 mg GAE/g extract in dry weight) obtained through USAE-EtOH from *Durvillaea incurvata* in another study, where a higher temperature (50 °C), a longer time (80.8 min), and a much higher ethanol concentration (70% *v*/*v*) were used [49]. It has been reported that phlorotannin extraction from brown seaweed begins to decrease with increasing ethanol concentration from 30 to 70% *v/v* [17]. Acoustic cavitation is the main extraction mechanism in USAE, so higher temperatures, higher co-solvent concentrations, and longer extraction times seem unnecessary [17,47].

Notably, the RP treatment resulted in a 1.9-fold and a 2.4-fold increase in TPC and TPhC, respectively (Figure 1A). These values for RE and ES were, on average, 2.3 times higher than those of CE (Figure 1A).

The antioxidant capacity was assessed by two distinct methodologies (DPPH and ORAC), considering that natural extracts present more than one antioxidant mechanism [50]. Overall, AC_ORAC_ showed higher values than AC_DPPH_ in all samples (Figure 1B). This difference may arise from several factors: the phenolic compounds extracted from *Durvillaea incurvata* frond could be more effective at scavenging peroxyl radicals than DPPH radicals; the methanolic medium utilized in the DPPH method might hinder the accessibility of phlorotannins to radicals; or the presence of certain compounds in the extract could interfere more with the DPPH method than the ORAC method [50,51,52]. The AC of CE was equivalent to DPPH and ORAC values previously reported for *Durvillaea incurvata* subjected to extraction by HPLE–glycerol and about twice the DPPH obtained by USAE-EtOH 70% [49], showing that high ethanol concentrations are detrimental for extraction efficiency. Incorporating RP led to significant enhancement of the AC, with AC_DPPH_ and AC_ORAC_ ES values ~1.5 and ~3.0 times higher than those of CE. On average, the AC values for ES and RE were similar, where DPPH_ES_ was lower than DPPH_RE,_ and ORAC_ES_ was higher than ORAC_RE_.

This suggests that RP selectively concentrated specific phlorotannins with higher reactivity toward the hydrogen atom transfer mechanism (DPPH assay) and peroxyl radicals (ORAC assay), leading to a significant increase in antioxidant capacity. Given the dose-dependent nature of phlorotannin bioactivity, it may be expected that this enhancement would translate into greater biological efficacy. In this sense, previous studies support this projection, as a purified *Durvillaea incurvata* extract exhibited a stronger α-glucosidase inhibitory effect than its crude counterpart [10]. Similarly, in diabetic mouse models, oral administration of 2,7″-phloroglucinol-6,6′-bieckol delayed carbohydrate absorption by inhibiting the relevant enzyme in a dose-dependent manner [53]. Additionally, research on the anti-inflammatory properties of seaweed extracts in mouse models found that purified eckol significantly reduced proinflammatory cytokine levels at the highest doses [54].

Overall, these findings indicate that RP improves the bioactivity of phlorotannin CE, potentially allowing lower doses to achieve comparable or enhanced biological responses.

### 3.2. Fourier Transform Infrared Spectroscopy Analysis

**Figure 2:**
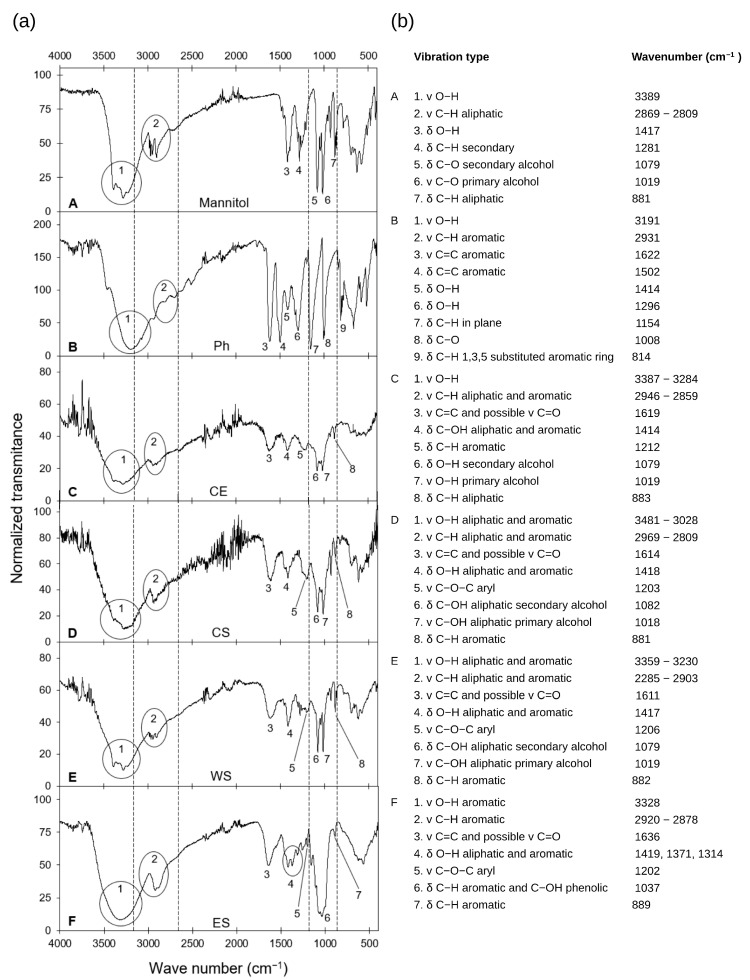
(**a**) FTIR spectra of mannitol, phloroglucinol, CE, and RP streams. (**b**) Characteristic bands of each FTIR spectrum associated with specific types of vibrations.

CE, all RP output streams, and standard mannitol and phloroglucinol solutions were analyzed using FTIR Spectroscopy. Analyzing the characteristic bands of all streams contributed to a better understanding of the USAE-RP’s selectivity (Figure 2).

In the six spectra, band 1 corresponding to O–H stretching was evident, which demonstrated the presence of groups characteristic of mannitol (aliphatic hydroxyl, Figure 2A,C–E) and polyphenols (aromatic hydroxyl, Figure 2B,F). Also, bands associated with aromatic rings (C–H stretching and C–H bending) were found in the six spectra. Specifically, FTIR spectra allowed the identification of several types of bonds commonly found in phlorotannins, including aryl ether linkages between aromatic rings that have been potentially associated with fucophloroethols [55]. Similarly, the FTIR spectrum of phloroglucinol enabled the detection of specific characteristic bands associated with this compound, and alterations in bands related to interferences, such as mannitol, were also evident for each analyzed sample.

The FTIR spectrum of CE (Figure 2C) showed an overlap of bands from mannitol and polyphenols, indicating the presence of both compounds. The low definition of a key band was attributed to residual water and ethanol from the USAE process. Additionally, a possible band of terrestrial polyphenols was detected (band 3 at 1619 cm^−1^, typically associated with C=O), while CS spectra revealed a significant amount of mannitol, which was expected due to the selectivity offered by the HP-20 resin. However, it also contained unadsorbed phlorotannins, possibly due to insufficient residence time or resin saturation. WS stream was rich in mannitol, while the ES lacked mannitol and was rich in phenolic compounds.

An important point should be discussed about the RP streams (Figure 2D–F). They all have a new band not detected on the phloroglucinol spectra (Figure 2B), indicating a new vibration associated with the C–O–C linkage. This band is characteristic of aromatic rings connected via ether bonds [56]. For instance, a sharp band associated with a C–O–C linkage (band 5) was observed in the spectrum of the elution stream (Figure 2F). Along with the broad O–H stretching band, it can be concluded that the elution stream is rich in phlorotannins.

### 3.3. Mannitol Content

Seaweed extracts contain undesirable compounds, such as mannitol, a predominant alcohol sugar of seaweeds [57]. Previously reported values for *Durvillaea incurvata* frond extracts ranged from 248 ± 39 to 310 ± 120 mg mannitol/g dried extract [10]. Here, the mannitol content of CE was 259 ± 24 mg mannitol/g dried extract, which falls within that range. However, the most interesting aspect is that the RP facilitated a substantial decrease in this constituent, with ES showing only 1% of the original mannitol value in CE. This low value indicates that applying USAE with a moderate concentration of EtOH, followed by RP, is quite effective for obtaining selective polyphenolic extracts from *Durvillaea incurvata* fronds with a reduced mannitol content [27]. Low mannitol content may enhance the value of the extract by increasing the concentration of polyphenols [58,59]. From a safety standpoint, the potential side effects associated with normal or elevated doses of mannitol (maximum dose: 1.5 g/kg/day, FDA) raise concerns regarding its use, as emphasized by relevant Food and Drug Administration guidelines. Therefore, future studies should focus on investigating the toxicity and side effects of sugar alcohols to assess their safety more accurately [60].

### 3.4. Tentative Identification of Phlorotannins and Determination of Their Relative Abundances by UHPLC-QToF Mass Spectrometry

The chemical identification of the phlorotannins present in the samples was initially based on a targeted analysis, which matched the identified phlorotannins with those previously reported [61]. Subsequently, a tentative identification of unreported phlorotannins was conducted. This tentative identification was based on matching the experimental molecular ions with the theoretical molecular weights of proposed phlorotannin structures, constructed according to the bonding types known for the different subfamilies (Fucols, Phloretols, Fucophlorethols, Fuhalols, Eckols) [62].

Five phlorotannins composed of 3 to 8 phloroglucinol units (PGUs) were proposed to be present in CE based on their [M − H]^−^ ions (Mass spectra in supplementary material, Figures S1–S5). The polymerization degree, molecular formula, fragmentations, and the associated errors in ppm for the found phlorotannins are shown in Table 1. The ionization conditions used in the MS method caused a first fragmentation at the source that produced phlorotannin fragments in MS^1^, i.e., it provided information similar to an MS^2^ analysis. In particular, fragmentation PGU + H_2_O + 2H^+^ was observed in several of the identified phlorotannins (Table 1). This fragmentation pattern, similar to that previously observed in a study on *Fucus vesiculosus*, indicated a possible fragmentation of the aryl–ether–aryl (C–O–C) bond [63]. Indeed, several identified phlorotannins exhibited comparable daughter ion fragmentation patterns linked to PGU + H_2_O + 2H^+^ with distinct molecular weights (Table 1), suggesting the presence of fucophloroethol isomers in the purification streams, which was confirmed due to the losses of PGU + H^+^ corresponding to aryl–aryl (C–C) bonds.

**Figure 3:**
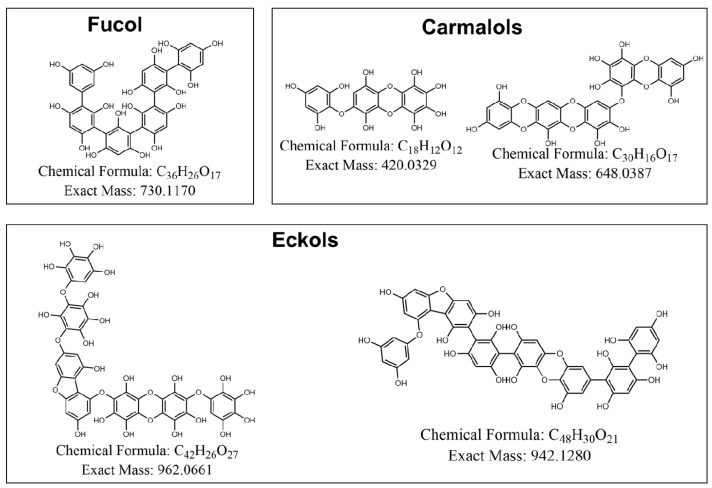
Proposed structures of the five identified phlorotannins in the extracts of *Durvillaea incurvata* frond.

Based on these aspects, a structural hypothesis is formulated for certain phlorotannins identified in brown seaweeds such as *Ascophyllum nodosum*, *Fucus vesiculosus*, and *Durvillea incurvata*, postulating breaks in the C–O–C and C–C bonds as the basic units of phloroglucinol. These tentative structures are categorized based on their type (Figure 3). It should be mentioned that the phlorotannins found in these extracts (carmalols, fucols, and eckols) are among those that have shown potent effects on the inflammatory response in previous studies [12,14,15].

**Figure 4:**
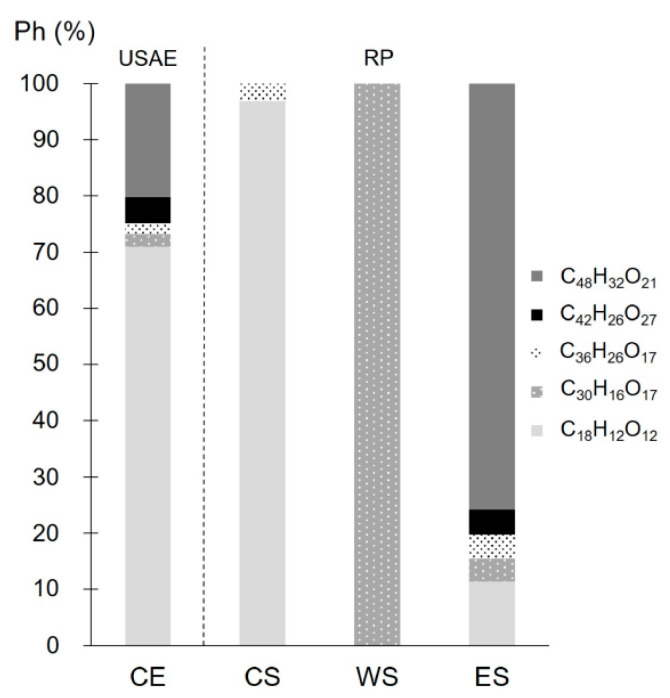
Distribution of the five proposed phlorotannins identified in the process streams (CE, CS, WS, and ES) from *Durvillaea incurvata* frond.

The five proposed phlorotannins identified in CE were also present in ES. The trimer and the hexamer were found in CS, while the pentamer was present in WS. Although the five CE phlorotannins were detected in ES, according to the relative areas, two compounds (C_18_H_12_O_12_ and C_30_H_16_O_17_) were more abundant in the intermediate RP streams (CS and WS, respectively) (Figure 4). The premature elution of these phlorotannins (C_18_H_12_O_12_ and C_30_H_16_O_17_) may be attributed to their smaller size, which reduces their residence time in the column relative to larger ones [27,64]. In this case, molecular size seems to play a more significant role than the high polarity of larger phlorotannins, which contain numerous hydroxyl groups that enhance their polarity and water affinity. It is important to consider these aspects when seeking extracts enriched in specific phlorotannins. Although ES typically exhibits the highest TPhC of the RP streams, its levels of certain target polyphenols can be low. Notably, each RP stream contained a phlorotannin with a relative abundance exceeding 70% (Figure 4), which is particularly relevant for targeted separation and has not been previously reported for RP purification of phlorotannins.

### 3.5. Process Evaluation: Quality Parameters and RP Mass Balance

**Figure 5:**
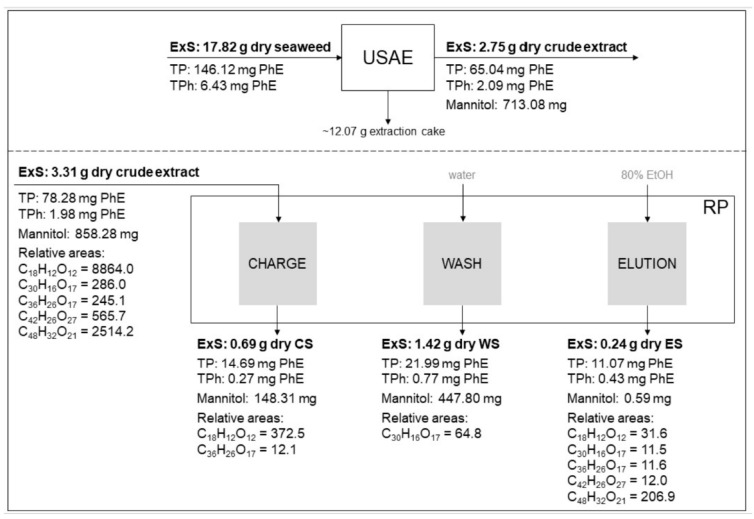
Relative mass balance of the integrated process of USAR-RP from *Durvillaea incurvata* frond. Relative abundances correspond to the ratio of the relative area of phlorotannin and ExS of the stream.

This complementary analysis provided a more comprehensive understanding of the separation process and allowed for a better assessment of its potential. The proportion of ExS generated by USAE was small, with the majority of solids remaining in the extraction cake (Figure 5). This was reflected in the *yield_ExS_*, which was 15%. Typically, the extraction yield from brown seaweed ranged between 10% and 37% when employing various extraction technologies, such as HPLE-EtOH, USAE-EtOH, and ASLE-EtOH [17,35,65,66]. Interestingly, the recoveries of polyphenols (Rec_TP_) and phlorotannins (Rec_TPh_) were significantly higher than the extraction yield (*yield_ExS_*), at 45% and 33%, respectively. This disparity highlights the potential for specific compounds to be extracted more efficiently than the overall yield might suggest [38]. Hence, both the extraction yield of solids and the recovery of specific bioactive compounds should be considered when evaluating extraction processes. While the first parameter offers insight into the overall extraction efficiency, the recoveries of polyphenols and phlorotannins may provide guidance on how to optimize the process for targeted extraction.

**Figure 6:**
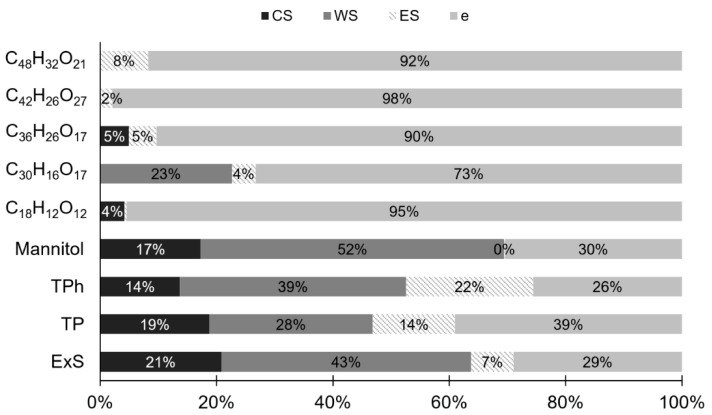
Percentage distribution of ExS, TP, TPh, Mannitol, and specific phlorotannins of CE in the RP streams.

The mannitol recovered in the CE stream was predominantly found in WS (52%, Figure 6), primarily containing polar compounds removed from the resin by water [38]. In contrast, ES contained a negligible amount of the mannitol originally present in CE, thereby achieving one of the main objectives of the RP process: the reduction or elimination of mannitol, a known undesirable compound in brown seaweed bioactive extracts [10,67]. The efficiency of mannitol removal by RP (*Eff_Ma_*) was ~100%, corroborating the findings mentioned above and indicating a good performance of the RP process. Due to mannitol removal, the TPC and TPhC in ES increased with respect to CE. However, the TP and TPh amounts in ES were significantly lower than in CE. This decrease is particularly marked in the relative areas of the five tentatively identified phlorotannins in the RP streams (Figure 5).

This behavior could be attributed to the RP, which distributed the TP and TPh present in CE among the three generated streams. Ideally, the proportions of TP and TPh in CS and WS should have been negligible. The presence of all four components in CS as compared to CE: ExS: 21%, TP: 19%, TPh: 14%, and Ma: 17% (Figure 6) suggests a possible overestimation of the CE loading rate, which may result in an insufficient residence time for complete adsorption of the target compounds. Alternatively, it could indicate early saturation of the resin in the RP system [38]. Readjustment of operational conditions could provide a viable solution to address these issues effectively. Although the operating conditions of the RP were not optimal, as the desired components (TP and TPh) were not exclusively found in ES, the RP still achieved a purification factor (*F_Purification_*) of ~2, indicating that the RP was effective in concentrating the target components. The polyphenol purity (*P_TP_*) in CE (~2%) was lower than that of RE (~5%). Nevertheless, through the RP, a *P_TP_* of ~5% was obtained in ES. Concurrently, both CS and WS exhibited a purity of around ~2%, likely due to the premature elution of phlorotannins.

The variation in AC reflected the trend observed in the *P_TP_* of the samples. Notably, both RE and ES, with a *P_TP_* of 5%, showed statistically equivalent AC_ORAC_ values. Likewise, CE, CS, and WS, with 2% purity, showed AC_ORAC_ values without significant differences.

The relative mass balance confirmed that the intermediate streams (CS and WS), which are typically discarded, contain significant proportions of ExS, TP, and TPh. Additionally, for these four components, the difference in relative mass between the input and output (*e*) ranged from 26% to 39%, suggesting that a significant proportion of phlorotannins may have been strongly adsorbed onto the resin, with their release likely occurring during resin regeneration. This loss is consistent with the characteristics of the HP-20 resin, which, despite having the highest adsorption capacity for phlorotannins compared to other macroporous resins, also exhibited the lowest desorption capacity [37]. When mass balance is applied to the relative areas of the five tentative identified compounds, *e* values showed a considerable increase ranging from 73% to 98%. These results would indicate the critical need to consider a deeper and more comprehensive chemical characterization to develop a precise process performance evaluation.

## 4. Conclusions

This study advances the development of sustainable technologies by demonstrating the effectiveness of combining USAE with RP to obtain mannitol-free purified extracts of phlorotannins from *Durvillaea incurvata* frond. The application of RP after USAE resulted in a significant enrichment of the crude extract, as evidenced by increased purities of both polyphenols and phlorotannins, resulting in a purification factor of ~2. The FTIR assay demonstrated the presence of phlorotannins and mannitol in the crude extract (CE) and intermediate RP streams (CS and WS), while no bands corresponding to this sugar alcohol were observed in the elution stream (ES). This finding confirms the phlorotannin selectivity of the USAE-RP process, which was also reflected in a 1.5-fold and 3-fold increase in their ORAC and DPPH antioxidant capacity, respectively, in the ES.

Furthermore, UHPLC-QToF allowed us to identify five new phlorotannins in *Durvillaea incurvata* frond USAE and RP extracts. The distribution of these phlorotannins in the different RP streams suggested that molecular size is the main factor that influences their separation. Future research should focus on optimizing RP’s operating conditions to enhance phlorotannin’s desorption and minimize target compound losses. The findings of this study lay a robust foundation for the scalable production of bioactive phlorotannin extracts, paving the way for more efficient and sustainable utilization of marine biomass in natural medical applications. To further optimize the integrated process in terms of biological activity, future studies should include cell-based assays. These will provide a more realistic evaluation of the bioactivity of the extracts, helping to refine their potential for therapeutic use.

## Figures and Tables

**Table 1:** Tentative identification of phlorotannins in the CE from *Durvillaea incurvata* frond using UHPLC-QToF-MS.

Polymerization Degree	Molecular Formula	Theoretical [M − H]^−^ (*m*/*z*)	Observed [M − H]^−^ (*m*/*z*)	Error (ppm)	MS^1^ (*m*/*z*)	MS^2^ (*m*/*z*)
Trimer	C_18_H_11_O_12_ (Carmalol)	419.0251	419.0241	2.3	389 (−30), 243 (−PGU + H_2_O + 2H^+^), 116 (PGU + H^+^)	N.D.
Pentamer	C_30_H_15_O_17_ (Carmalol)	647.0309	647.0368	−9.1	535 (111), 389 (PGU + H2O + 2H^+^), 243 (PGU + H2O + 2H^+^), 116 (PGU + H^+^)	116, 100 (–O)
Hexamer	C_36_H_25_O_17_ (Fucol)	729.1092	729.1088	0.5	681 (47), 535 (−PGU + H_2_O + 2H^+^), 389 (−PGU + H_2_O + 2H^+^), 243 (−PGU + H_2_O + 2H^+^), 116 (PGU + H^+^)	A) 116, 100 (–O)
Heptamer	C_42_H_25_O_27_ (Eckol)	961.0583	961.0578	0.6	815 (145), 681 (134), 535 (−PGU + H_2_O + 2H^+^), 389 (−PGU + H_2_O + 2H^+^), 243 (−PGU + H_2_O + 2H^+^), 116 (PGU + H^+^)	N.D.
Octamer	C_48_H_31_O_21_ (Eckol)	943.1358	943.1305	5.6	791 (151), 501 (−2PGU + H_2_O + 2H^+^), 389 (112), 243 (−PGU + H_2_O + 2H^+^), 116 (PGU + H^+^)	A) 791, 225 (566), 167 (60), 81 (84)

N.D., non-detected; PGU, phloroglucinol units.

## Data Availability

The data was provided in Supplementary Materials.

## Supplementary Materials

The following supporting information can be downloaded at: https://www.mdpi.com/article/10.3390/antiox14030250/s1. Figure S1: Mass Spectra (MS^1^) of C_18_H_11_O_12_ in crude extract using UHPLC-QToF, Figure S2: Mass Spectra of C_30_H_15_O_17_ in crude extract using UHPLC-QToF: A) MS^1^ and B) MS^2^, Figure S3: Mass Spectra of C_36_H_25_O_17_ in crude extract using UHPLC-QToF: A) MS^1^ and B) MS^2^, Figure S4: Mass Spectra (MS^1^) of C_42_H_25_O_27_ in crude extract using UHPLC-QToF, Figure S5: Mass Spectra of C_48_H_31_O_21_ in crude extract using UHPLC-QToF: (A) MS^1^ and (B) MS^2^.
