# Supplementary material for: Sustainable Recovery of Phlorotannins from Durvillaea incurvata: Integrated Extraction and Purification with Advanced Characterization
Source: Antioxidants (Basel). 2025 Feb 21;14(3):250. doi: 10.3390/antiox14030250 (PMC11939385; doi:10.3390/antiox14030250)
Supplement: Supplementary file 1 [file antioxidants-14-00250-s001.zip › antioxidants-3435688-supplementary.pdf]

Sustainable Recovery of Phlorotannins from *Durvillaea incurvata*: Integrated Extraction and Purification with Advanced Characterization

Pamela Raquel Rivera-Tovar [1], Gabriela Contreras-Contreras [1], Paulina Isabel Rivas-Reyes [2], Jara Pérez-Jiménez [3,4], Maximiliano Martínez-Cifuentes [5], José Ricardo Pérez-Correa [6] and María Salomé Mariotti-Celis [1,*]

[1] Nutrition and Dietetics School, Faculty of Medicine, Universidad Finis Terrae, Pedro de Valdivia 1509, Providencia, Santiago 7501015, Chile; privera@uft.cl (P.R.R.-T.); gabriela.contreras@ug.uchile.cl (G.C.-C.)
[2] Food Technology Section, Department of Analytical Chemistry, Nutrition and Food Science, School of Veterinary Sciences, University of Santiago de Compostela, 27002 Lugo, Spain; privasr@uft.edu
[3] Department of Metabolism and Nutrition, Institute of Food Science, Technology and Nutrition (ICTAN-CSIC), 28040 Madrid, Spain; jara.perez@ictan.csic.es
[4] CIBER de Diabetes y Enfermedades Metabólicas Asociadas (CIBERDEM), Instituto de Salud Carlos III (ISCIII), 28029 Madrid, Spain
[5] Departamento de Química Orgánica, Facultad de Ciencias Químicas, Universidad de Concepción, Edmundo Larenas 129, Concepción 4070371, Chile; maxmartinez@udec.cl
[6] Chemical and Bioprocess Engineering Department, School of Engineering, Pontificia Universidad Católica de Chile, Vicuña Mackenna 4860, Macul, Santiago 7820436, Chile; jperezc@uc.cl
* Correspondence: mmariotti@uft.cl

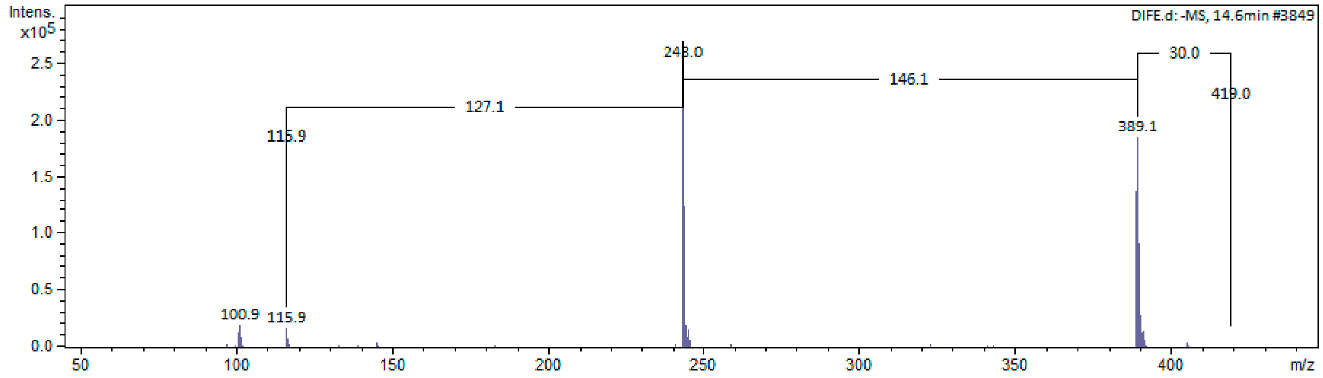

Figure S1. Mass Spectra (MS^1) of $C_{18}H_{11}O_{12}$ in crude extract using UHPLC-QToF.

A)

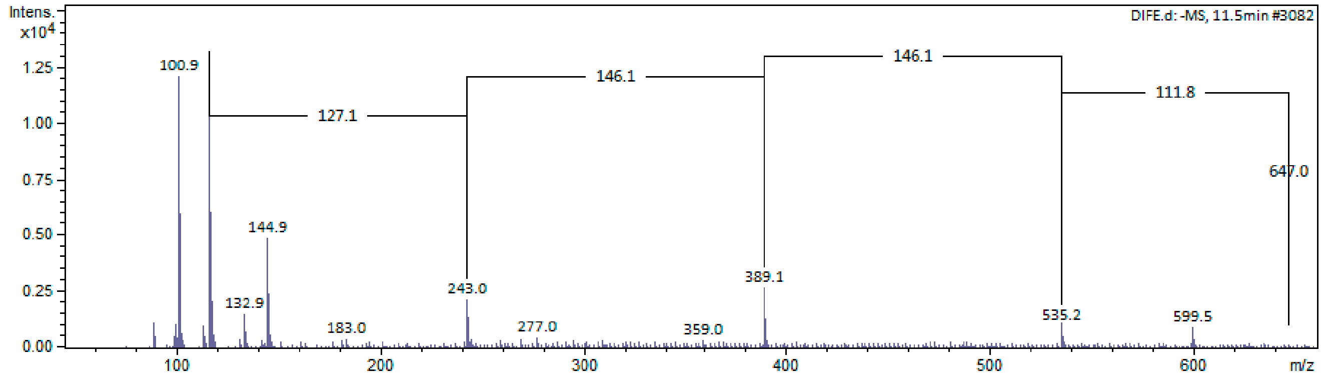

B)

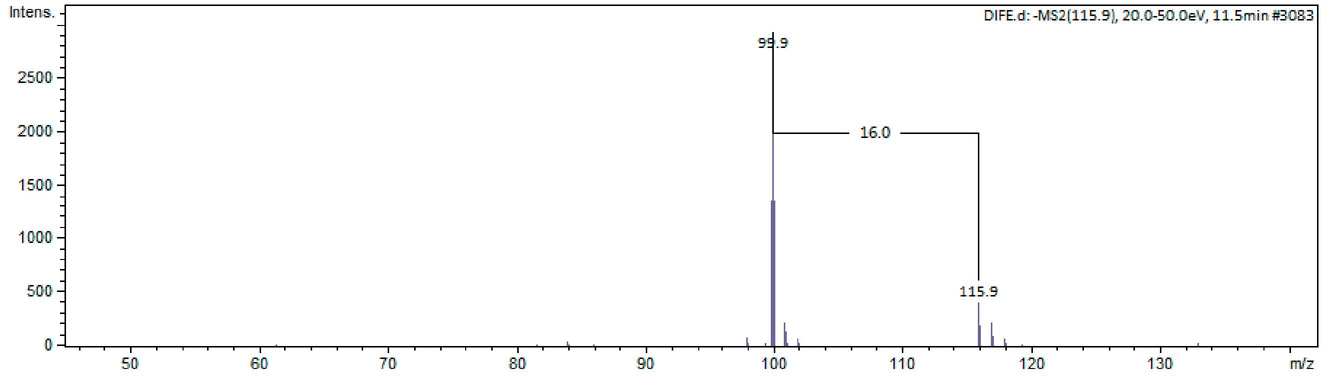

Figure S2. Mass Spectra of $C_{30}H_{15}O_{17}$ in crude extract using UHPLC-QToF: A) MS^1 and B) MS^2.

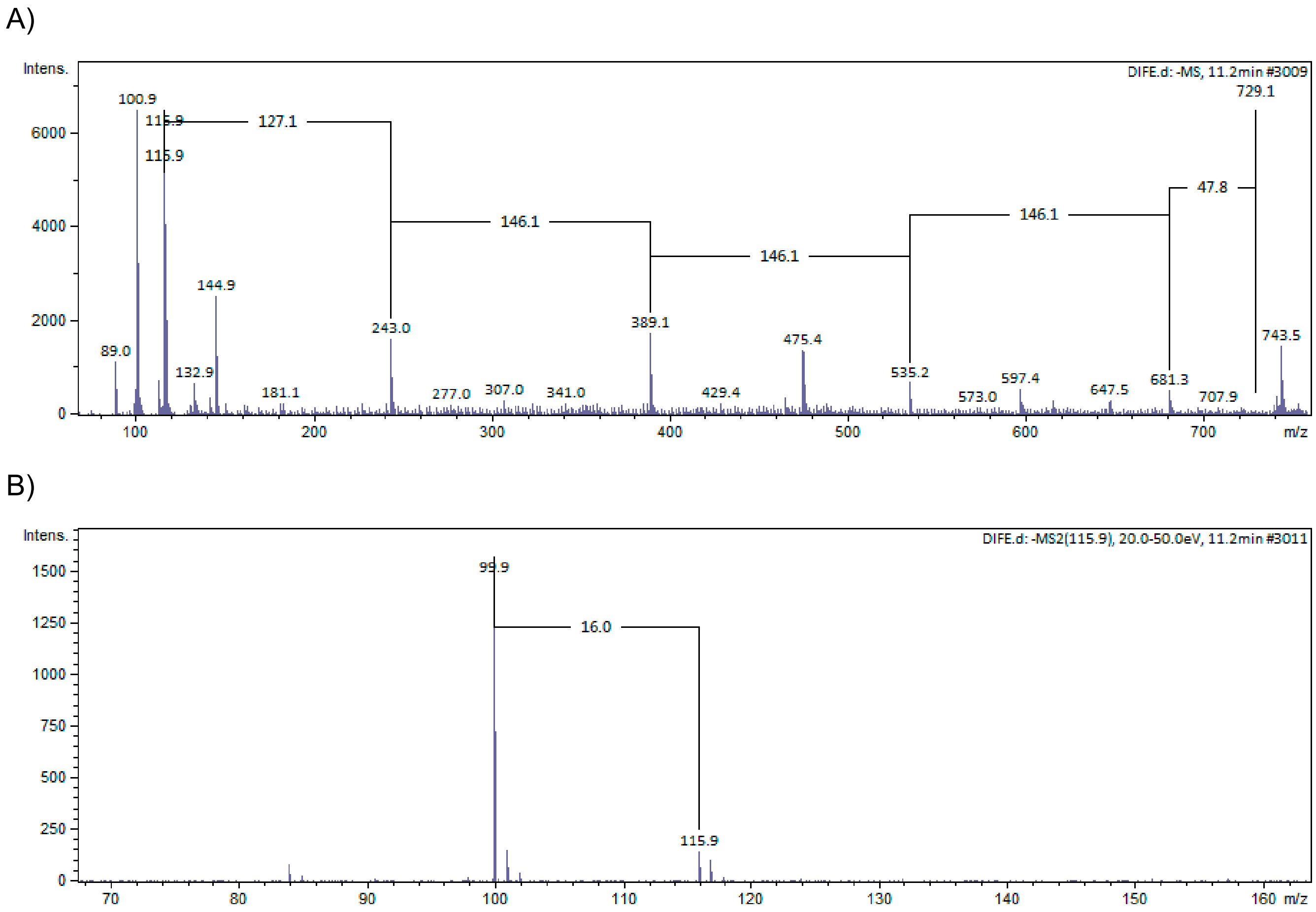

Figure S3. Mass Spectra of $C_{36}H_{25}O_{17}$ in crude extract using UHPLC-QToF: A) MS^1 and B) MS^2.

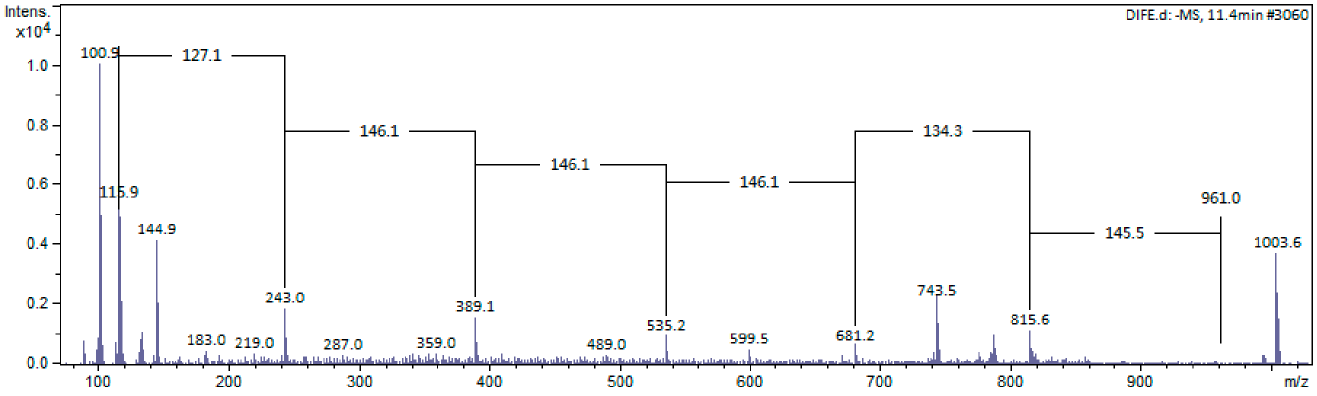

Figure S4. Mass Spectra (MS^1) of $C_{42}H_{25}O_{27}$ in crude extract using UHPLC-QToF.

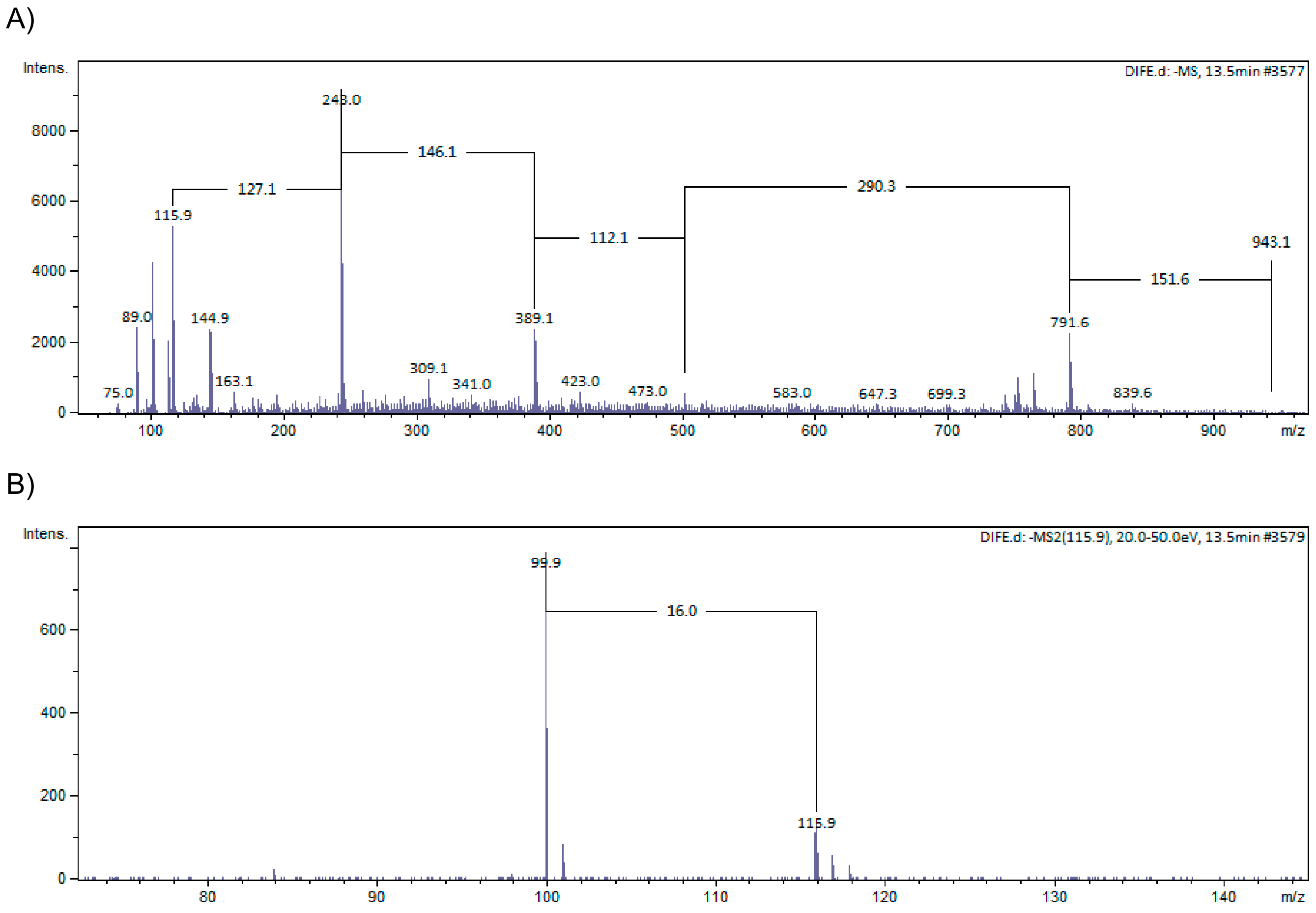

Figure S5. Mass Spectra of $C_{48}H_{31}O_{21}$ in crude extract using UHPLC-QToF: A) MS^1 and B) MS^2.
